# Identifying risk factors for severe omicron infection in allogeneic hematopoietic stem cell transplant recipients with hematologic malignancies

**DOI:** 10.1002/cnr2.2103

**Published:** 2024-06-21

**Authors:** Zhihui Li, Lei Wang, Qinlong Zheng, Teng Xu, Keyan Yang, Xianxuan Wang, Xiaopei Wen, Caiyan Zhang, Jingjing Wang, Yanzhi Song, Yongqiang Zhao, Xiaoyu Zheng, Tong Wu

**Affiliations:** ^1^ Department of Bone Marrow Transplantation Beijing GoBroad Boren Hospital Beijing China; ^2^ Department of Medical Laboratory Beijing GoBroad Boren Hospital Beijing China; ^3^ Gobroad Research Center Gobroad Healthcare Group Beijing China

**Keywords:** Allo‐HSCT, hematologic malignancies, risk factors, SARS‐CoV‐2 omicron

## Abstract

**Background:**

In December 2022, a large‐scale epidemic occurred in China due to Omicron variant of SARS‐CoV‐2. This study explored risk factors for Omicron infection in transplant recipients at our institution and investigated the factors influencing the severity of SARS‐CoV‐2 Omicron infection among recipients of allo‐HSCT.

**Methods:**

This single‐center study investigated totally 63 allogeneic hematopoietic stem cell transplant patients infected with Omicron variant at the Beijing GoBroad Boren Hospital Transplant Center during December 2022 and analyzed their risk factors.

**Results:**

The study included 63 allogeneic hematopoietic stem cell transplant patients who developed Omicron infection. There were 34 mild and 29 moderate to severe cases. Their median age was 22 years (range, 1–65 years), with the male‐to‐female ratio being 1:1.1. Acute myeloid leukemia (53.97%), acute lymphoblastic leukemia (42.86%), and non‐Hodgkin lymphoma (3.17%) were underlying diseases. The median time between HCT and Omicron infection was 8.45 months. Significant predictive factors for moderate to severe Omicron infection included older age (*p* < .0001), cGVHD (*p* = .0195), concurrent bacterial infection (*p* < .0001), low absolute lymphocyte count (*p* = .026), low CD4/CD8 ratio (*p* = .0091), high CRP (*p* < .0001), high serum ferritin (*p* = .0023), high D‐dimer (*p* < .0001), low CD4 absolute count (*p* = .0057), and low B‐cell absolute count (*p* = .0154). A moderate to high HCT‐CI score tended to be associated with moderate to severe infection (*p* = .0596).

**Conclusion:**

This study indicates that risk factors for severe Omicron infection include certain clinical characteristics, such as age, cGVHD, and inflammatory response.

**Abbreviations**
aGVHD: acute graft‐versus‐host disease
ALL: acute lymphoblastic leukemia
AML: acute myeloid leukemia
ARDS: acute respiratory distress syndrome
BP: biological processes
BU: busulfan
cGVHD: chronic graft‐versus‐host disease
CI: confidence interval
CIBMTR: center for international blood and marrow transplant
COVID‐19: corona virus disease 2019
EBV: Epstein–Barr virus
HCT‐CI: hematopoietic cell transplantation‐comorbidity index
HSCT: hematopoietic stem cell transplant
IFN‐γ: interferon‐γ
IGV: integrative genomics viewer
IST: immunosuppressive therapy
MAF: minor allele frequency
NHL: non‐Hodgkin lymphoma
NIH: National Institute of Health
SARS‐COV‐2: severe acute respiratory syndrome coronavirus 2
TBI: total body irradiation
TDM: therapeutic drug monitoring

## 1 INTRODUCTION

Since 2020, SARS‐CoV‐2 has spread rapidly, causing a pandemic.^1^, ^2^ In the early stages of COVID‐19 pandemic, hematopoietic stem cell transplant (HSCT) recipients, particularly those with malignant hematologic diseases, experienced high mortality and severe COVID‐19 rates owing to immune deficiencies caused by their underlying diseases.^3^, ^4^, ^5^ During the ongoing spread and mutation of this virus, the SARS‐CoV‐2 Omicron variant emerged, exhibiting strong immune escape and transmissibility but reduced rates of severe disease, hospitalization, and death.^6^ Previous studies have investigated the clinical outcomes of Omicron infection in immunocompromised patients^7^; however, there are very rare reports on the outcomes of HSCT patients. In addition, limited information is available on the incidence, infection process, and prognostic factors of the Omicron variant in transplant patients. Due to the influence of the pandemic, several published case reports and cohort analyses have indicated that this specific patient population has a higher rate of infection and complications. Following HSCT, COVID‐19 patients experience more rapid disease progression and greater invasiveness and are more prone to develop severe complications, causing poor prognosis.^8^, ^9^ By investigating the clinical characteristics of the SARS‐CoV‐2 Omicron variant infection among HSCT patients, it may be possible to more accurately identify patients who have poor prognosis at an early stage and manage them more effectively to improve outcomes.

Due to the urgent need for clinical experience to guide management of this new disease, we describe the clinical characteristics of COVID‐19 in the single‐center HSCT recipient population in the widespread community transmission wave caused by the SARS‐CoV‐19 Omicron variant. Through analyzing inflammation levels, peripheral blood lymphocyte subgroups and plasma cytokine levels during the infection process, we aimed to explore factors influencing severity of SARS‐CoV‐2 Omicron variant infection among allo‐HSCT patients.

## 2 METHODS

### 2.1 Patient setting

This study retrospectively investigated medical records from 63 consecutive SARS‐CoV‐2‐positive HSCT recipients at our institution between December 16, 2022, and February 25, 2023, and performed descriptive statistical data analysis. Patients were enrolled during COVID‐19 diagnosis and followed up until February 25, 2023. Disease‐related characteristics, radiological features, laboratory test result values, antiviral drug use, baseline immunosuppression management, and transplant outcomes of the patients were collected using standardized case report forms.

All patients had been seen at outpatient visits or hospitalized and had symptomatic signs suggestive of COVID‐19. They were all tested for SARS‐CoV‐2 infection. COVID‐19 was diagnosed via real‐time reverse transcription polymerase chain reaction (RT‐PCR) or novel coronavirus antigen detection using nasopharyngeal swab or sputum samples. All suspected or confirmed SARS‐CoV‐2‐infected HSCT recipients were placed on droplet and contact precautions, involving personal protective equipment as required by institutional regulations.

### 2.2 Data collection

According to the grading criteria provided by the NIH COVID‐19 Clinical Practice Guidelines, the patients were categorized as having mild, moderate, severe, and critical cases based on imaging results, SpO_2_, SpO_2_/FiO_2_, hypotension, acute respiratory distress syndrome (ARDS), presence of respiratory failure, septic shock, and/or multiorgan dysfunction. Adverse outcomes were set as progressive respiratory failure (i.e., persistent worsening of SpO_2_), continuous progression of ARDS, admission to intensive care unit (ICU), and/or death. Factors influencing critical illness due to SARS‐CoV‐2 infection were assessed based on available laboratory parameters (C‐reactive protein, white blood cell count, lymphocyte count, neutrophil count, lymphocyte subpopulation ratios, cytokine levels, and soluble CD25 levels). The standardized HCT comorbidity index (HCT‐CI) was adopted for reporting comorbidities.^10^

Disease duration was set as the time from SARS‐CoV‐2 infection diagnosis to the recorded resolution of infection or death. Infection status was considered persistent when no clinically significant improvement signs existed or resolved if all signs and symptoms were resolved and the patient had completed the planned course of infection treatment.

### 2.3 Statistical analysis

Descriptive statistical methods were used to compare baseline demographic characteristics. Data analysis of binary variables was conducted with chi‐square and Fisher‐exact tests. Multinomial variables were explored with Cochran–Armitage trend tests and Fisher‐exact tests. Using *t* tests and rank sum tests, continuous variables were studied. SAS 9.4 software was adopted for the statistical analyses.

## 3 RESULTS

### 3.1 Baseline characteristics of the patients

As of February 25, 2023, totally 63 HSCT recipients after the SARS‐CoV‐2 Omicron variant infection were included, with mild (54.0%), moderate (31.7%), severe (9.5%), and critical (4.76%) severities (Table 1). The median age was 22 years (range: 1–65 years), and the male‐to‐female ratio was 1:1.1. Underlying diseases were acute myeloid leukemia (50.79%), acute lymphoblastic leukemia (42.86%), and non‐Hodgkin lymphoma (6.35%). The median time between HCT and Omicron infection was 8.45 months (range: 0.26 to 208.18 months). Sixteen patients (25.4%) became infected with Omicron within 100 days after HCT. All the involved patients received myeloablative preconditioning. Donor types included haploidentical (80.95%), sibling matched (15.87%), and unrelated (3.17%). Previous history of grade II‐IV acute graft‐versus‐host disease (GVHD), cGVHD, and the current use of immunosuppressive therapy (IST) were reported in 20.6%, 42.9%, and 87.3% of the patients, respectively. Concurrent bacterial infections were found in 11% of the patients. Among the 63 SARS‐CoV‐2‐infected HSCT recipients, only seven had received inactivated SARS‐CoV‐2 vaccines, with four receiving two doses and three receiving only one dose. The last vaccination was more than a year before the infection.

**TABLE 1:** Baseline characteristics of HCT recipients diagnosed with COVID‐19.

Characteristic	Total (*N* = 63)	COVID‐19 severity		*p* value
		Mild (*N* = 34)	Moderate–severe (*N* = 29)	
Age, year, median (range)	22 (1–65)	10 (1–58)	36 (6–65)	<.001
Male sex, *n* (%)	30 (47.62)	15 (44.12)	15 (51.72)	.547
Hematologic malignancy, *n* (%)				
ALL	27 (42.86)	15 (44.12)	12 (41.38)	.146
AML	32 (50.79)	15 (44.12)	17 (58.62)	
NHL	4 (6.35)	4 (11.76)	0 (0.00)	
Donor type, *n* (%)				
Haplo	51 (80.95)	28 (82.35)	23 (79.31)	1.000
Matched sibling (identical)	10 (15.87)	5 (14.71)	5 (17.24)	
Matched unrelated	2 (3.17)	1 (2.94)	1 (3.45)	
Conditioning, *n* (%)				
TBI	32 (50.79)	15 (44.12)	17 (58.62)	.251
BU	31 (49.21)	19 (55.88)	12 (41.38)	
HCT comorbidity index, *n* (%)				
Low risk	50 (79.37)	30 (88.24)	20 (68.97)	.060
Medium and high risk	13 (20.63)	4 (11.76)	9 (31.03)	
Prior acute GVHD grade II–IV, *n* (%)	13 (20.63)	5 (14.71)	8 (27.59)	.208
Prior chronic GVHD, *n* (%)	27 (42.86)	10 (29.41)	17 (58.62)	.020

Abbreviations: BU, busulfan; CART, chimeric antigen receptor‐T cell therapy; COVID‐19, coronavirus disease 2019; GVHD, graft‐versus‐host disease; HCT, hematopoietic stem cell transplant; TBI, total body irradiation.

### 3.2 Treatment after SARS‐CoV‐2 infection

Symptomatic treatment with medications was administered to patients with symptoms restricted to the upper respiratory tract, the normal blood oxygen saturation, and no radiological features of pneumonia, according to COVID‐19 Clinical Practice Guidelines provided by the NIH.^11^ Corticosteroids, tocilizumab, and/or JAK2 inhibitors were added in cases of progressive respiratory failure and increased inflammatory parameters. If bacterial or fungal coinfection was suspected, empirical antibiotic treatment was given.

Management of immunosuppression was determined in consultation with hematopoietic stem cell transplant physicians and pulmonology physicians but usually involved gradual discontinuation or reduction of maintenance therapy. Calcineurin inhibitors were temporarily discontinued or reduced when Paxlovid (nirmatrelvir/ritonavir) was initiated, followed by close therapeutic drug monitoring (TDM) thereafter, paying attention to Paxlovid (nirmatrelvir/ritonavir) drug interactions. Patients with COVID‐19 were given 0.5–1 mg/kg methylprednisolone for anti‐inflammatory treatment, depending on the severity of their lung lesions. Some patients with severe pneumonia manifestations, including hypoxemia, received increased doses of corticosteroids.

This cohort of patients was in complete remission when they were infected with COVID‐19, and all of their bone marrow status was complete donor type. We also did not observe an effect of COVID‐19 infection on the chimerism rate.

### 3.3 Severity of omicron infection

Fifty‐four percent of the infected individuals had mild symptoms, while 46% showed moderate to severe symptoms. Clinical manifestations included pneumonia or abnormal chest imaging (46%), hypoxemia (17%), ICU admission (4.76%), and mechanical ventilation (4.7%). Treatment approaches were nirmatrelvir/ritonavir (19%), molnupiravir (11%), azvudine (4.76%), corticosteroids (42.9%, including different types of steroids, such as methylprednisolone, dexamethasone, and prednisone), and ruxolitinib (42.9%).

The median time of viral nucleic acid negativity was 12 (3–46) days, and two patients remained viral nucleic acid positive for 2 months (Table 2). By February 25, 2023, with the median follow‐up of 70 (14–89) days, three deaths were reported at 14, 34, and 44 days after Omicron infection, and two of them had progressive disease. No Omicron reactivation was found in the patients developing mild illness, while 33% reactivation was observed in the patients exhibiting more severe patterns (moderate, severe, and critical). The median time to viral clearance was 11 (range, 3–32) days in patients with mild disease and 13 days (range, 6–46 days) in those with moderate to severe disease.

**TABLE 2:** Clinical characteristics of HCT recipients diagnosed with COVID‐19.

Characteristic	Total (*N* = 63)	COVID‐19 severity		*p* value
		Mild (*N* = 34)	Moderate–severe (*N* = 29)	
Time since HCT, month, median (range)	8.45 (0.26–208.18)	9.24 (0.26–208.18)	6.97 (0.89–30.84)	.285
COVID‐19 diagnosis in first 100 d post‐HCT, *n* (%)	16 (25.4)	8 (23.53)	8 (27.59)	.712
Current IST, *n* (%)	21 (36)	5 (17)	8 (40)	<.001
Prednisone ≥ 20 mg/d	5 (9)	2 (7)	0	
Prednisone <20 mg/d	9 (16)	1 (3)	5 (25)	
Tacrolimus ± prednisone	12 (21)	2 (7)	6 (30)	
Ruxolitinib ± prednisone	5 (9)	1 (3)	1 (5)	
Concurrent infections, *n* (%)	48 (76.19)	21 (61.76)	27 (93.1)	.004
Lab indices at COVID diagnosis, median				
WBC, g/dL	3.95	3.96	3.63	.321
Neutrophils, K/mL	1.87	1.82	1.87	.989
Lymphocytes, K/mL	1.01	1.56	0.84	.026
C‐reactive protein, mg/dL	4.50	0.75	14.55	<.001
D‐dimer	0.64	0.25	1.03	<.001
Ferritin, ng/dL	2260.10	1255.85	2734.50	.002
B lymphocyte ratio	0.04	0.05	0.03	.076
B‐cell absolute count	0.04	0.07	0.02	.0154
CD4 absolute count, and low	0.20	0.26	0.11	.0057
CD3 ratio	0.80	0.74	0.85	.005
CD4/CD8 ratio	0.37	0.47	0.28	.009
IL6	7.67	7.26	11.81	.396
IL8	11.11	9.45	12.91	.725
TNF‐α	1.97	2.18	1.84	.141
sCD‐25	268.67	270.94	262.75	.725
Follow‐up after COVID‐19, month, median (range)	2.30 (0.46–2.93)	2.30 (1.55–2.93)	2.27 (0.46–2.53)	.423

Abbreviations: CART, chimeric antigen receptor‐T cell therapy; COVID‐19, coronavirus disease 2019; GVHD, graft‐versus‐host disease; HCT, hematopoietic stem cell transplant; IST, immunosuppressive therapy.

### 3.4 Significant risk factors for moderate to severe omicron infection

Older age (*p* < .0001) (Table 1), low absolute lymphocyte count (*p* = .026), low C4/CD8 ratio (*p* = .009), high CD3 ratio (*p* = .005), elevated CRP levels (*p* < .0001), high serum ferritin (*p* = .002), high D‐dimer (*p* < .001), low CD4 absolute count (*p* = .0057), low B‐cell absolute count (*p* = .0154), and high current IST(*p* < .001) (Table 2).

Significant predictors for COVID‐19 severity from univariate logistic regression model (mild vs. moderate–severe COVID‐19) including gene mutation related to autoimmune disease (*p* = .009 OR = 6.10 95% CI: 1.46, 25.38), presence of chronic GVHD (*p* = .0195, OR = 3.4, 95% CI: 1.1967, 9.6596), concurrent bacterial infection (OR = 15.9, 95% CI: 3.9706, 63.5821, *p* < .0001), patients with CR before HSCT (*p* = .073, OR = 3.38, 95% CI: 0.12, 1.12), and moderate to high HCT‐CI scores tended to have moderate to severe infection (*p* = .0596, OR = 3.375, 95% CI: 0.9137, 12.4664) (Table 3).

**TABLE 3:** Fisher‐exact or chi‐square analysis of factors associated with disease severity in HCT recipients diagnosed with COVID‐19.

Characteristic	COVID‐19 severity (mild vs. moderate–severe)	
	OR (95% CI)	*p* value
Concurrent bacterial infection	15.89 (3.97–63.58)	<.0001
Chronic GVHD	3.40 (1.20–9.66)	.020
HCI‐CI medium and high risk versus low risk	3.38 (0.91–12.47)	.060
CR before HSCT	0.37 (0.12–1.12)	.073

*Note*: Not significant: Gender (*p* = .547), EMD (*p* = .681), chemotherapy before HSCT (*p* = 1.000), relapse before HSCT (*p* = .758), CART (*p* = .405), conditioning (BU vs. TBI) (*p* = .251), second HSCT (*p* = .689), acute GVHD (*p* = .422), fungus infection (*p* = .721), virusemia (*p* = .521), relapse after HSCT (*p* = .777), relapse in 3 months (*p* = 1.000), CR versus non‐CR at infection (*p* = .280), CR versus non‐CR after HSCT (*p* = .280).

Abbreviations: BU, busulfan; CART, chimeric antigen receptor‐T cell therapy; COVID‐19, coronavirus disease 2019; HCT, hematopoietic stem cell transplant; TBI, total body irradiation.

## 4 DISCUSSION

This retrospective study investigated the real‐world effect of the SARS‐CoV‐2 Omicron variant infection on recipients of allo‐HSCT treatment after the promotion of anti‐COVID‐19 drugs including Paxlovid (nirmatrelvir/ritonavir) and molnupiravir. In previous studies targeting SARS‐CoV‐2, the mortality rate was higher among those after infection with the original strain of SARS‐CoV‐2. In a US cohort of 34 HCT recipients, Varma et al.^12^ presented the COVID‐19 mortality rate of 21%, with the majority of deaths occurring in the allogeneic HCT group (25%). Similarly, Sharma et al.'s analysis from the Center for International Blood and Marrow Transplant Research (CIBMTR) and Shah et al.'s retrospective report from New York indicated an allogeneic HCT mortality rate of 22% with higher severe COVID‐19 and mechanical ventilation requirements compared with autologous hematopoietic stem cell transplant recipients.^13^, ^14^ The overall mortality of patients in our cohort, where only a minority had been vaccinated, was 4.76%. This might be due to the use of antiviral drugs or infection caused by different SARS‐CoV‐2 strains. However, further studies are warranted due to the different vaccine backgrounds of the patients in this cohort study, further studies may be needed.^15^, ^16^

Important risk prediction factors for severity of COVID‐19 included different levels of immune cells, inflammatory factors, D‐dimer levels, cGVHD, and bacterial infection. In HCT recipients, factors significantly increasing the mortality rate compared with the general population were multifaceted, mainly due to severe immune dysregulation, long‐term immune suppression, GVHD, and reduced blood cells.^17^

In early research, Mathew et al. defined three immune types of hospitalized COVID‐19 patients^1^: those with strong CD4+ T‐cell activation and proliferation, positively correlating with disease severity^2^; those with weak CD8+ T‐cell, CD4+ T‐cell, and memory B‐cell responses, not relating to disease severity; and^3^ those lacking detectable lymphocyte responses, negatively associating with disease severity.^18^ Low absolute lymphocyte count (*p* = .026), low CD4+ T/CD8+ T ratio (*p* = .0091), high CD3+ T‐cell ratio (*p* = .0062), low CD4 absolute count (*p* = .0057), and low B‐cell absolute count (*p* = .0154) all suggested poor prognosis. Activated CD4+ T cells in COVID‐19 patients are mainly cytotoxic T‐helper follicular cells and cytotoxic T‐helper cells, which kill B cells, inhibit germinal center reactions, delay antibody production, and worsen the disease.^19^ Previous studies in China also found that a reduction in B‐cell levels is closely related to delayed clearance of the virus, possibly due to the increased proportion of plasmablasts in peripheral blood, influencing humoral immune function and reducing the generation rate of specific antibodies.^20^ In follow‐up research, it was shown that the reduction in CD4+ T/CD8+ T ratio was relatively stable in both healthy individuals and recovering patients and did not change gradually with recovery from the disease.^21^ Therefore, this indicator can more specifically identify high‐risk groups that may experience progression to severe illness, especially in people over 50 years old. In addition, some studies also suggest that the CD4+ T/CD8+ T ratio can be a vital identification indicator for severe COVID‐19 and the need for ICU support treatment.^22^, ^23^

Previous studies indicated that there was no obvious relationship between HCT‐CI and survival. However, in our retrospective study, HCT‐CI scores for patients with medium to high risk were associated with a trend toward moderate to severe infection. This finding is inconsistent with the conclusions drawn by Sharma and Mushtaq et al.^13^, ^24^; it may be caused by a relatively smaller sample size in previous studies or differences in viral infection subtypes; therefore, further extensive statistical analysis studies are needed for validation.

As reported in previous studies, our center's statistical results indicate that high serum ferritin levels showed a relationship to COVID‐19 severity and mortality.^5^, ^14^ However, there is still controversy on the correlation between elevated CRP level and poor prognosis. Mushtaq's study found no clear correlation between high CRP levels and poor prognosis,^24^ while others believe that high CRP levels usually indicate poor prognosis.^25^ Our center's research suggests that high C‐reactive protein predicts poor prognosis for COVID‐19 patients, which may be associated with the use of baseline immunosuppressive drugs in our center, causing the index to remain within a relatively normal range at the onset of the disease. Abnormalities in inflammatory factors appear only when the disease worsens, and the inflammatory state at the onset of the disease is already at a high level, indicating severe illness and poor prognosis.

In a previous single‐center study on pediatric HSCT recipients, Averbuch et al. found that HSCT recipients with chronic GVHD, especially those concurrently using immunosuppressive drugs including mycophenolate mofetil (MMF), were more probably to develop severe or critical illness after SARS‐CoV‐2 infection.^26^ This may be resulted from the concurrent use of immunosuppressive drugs, a higher proportion of medium‐risk immune deficiency scores, and a higher likelihood of coinfection with other pathogens. In a multicenter study led by Sharma, the use of immunosuppressive drugs within 6 months before SARS‐CoV‐2 infection exerted no clear effect on the prognosis of COVID‐19.^13^ However, further subgroup analyses were not possible due to the small sample size. Some studies from other countries suggest that poor prognosis in such patients is related to factors including low immune response and low antibody titers after vaccination.^27^ Nevertheless, in our center's study, most patients did not receive a full course of vaccination, and there were some differences compared with other studies. The efficiency of the immune response requires further analysis.

In addition to above‐mentioned risk factor analysis, statistical analysis was performed on the vaccination status of patients at our center. Among the 63 HSCT recipients infected with SARS‐CoV‐2, only seven had received the SARS‐CoV‐2 inactivated vaccine, with four receiving two doses of the vaccine and three receiving just one dose. The last vaccination was more than 1 year before the infection. During the 3‐year COVID‐19 pandemic, the protective effect of vaccines for susceptible populations has been widely recognized in reducing infections and mortality rates.^28^ Studies have shown that vaccinated HSCT recipients have relatively better prognosis but that those who have not been vaccinated at the time of infection usually have more severe disease. In this immunocompromised population, encouraging vaccination can benefit HSCT recipients when they become infected with SARS‐CoV‐2.^29^ However, with the emergence of variant strains, the protective efficiency of existing vaccines has declined to varying degrees, and some studies even suggest that the antibody titer against the original strain increases significantly in people who have obtained three doses of inactivated vaccine but that the specificity of the antibody titer against Omicron decreases.^30^ Typically, the effectiveness of SARS‐CoV‐2 infection, hospitalization, and mortality rates declines over time, with Omicron variant baseline vaccine effectiveness levels being significantly lower than those for other variants. In cancer patients, particularly those with hematological malignancies, breakthrough infections are more probably to occur than in the general population, and this group of patients usually has a higher probability of developing severe and critical illness.^31^ Therefore, other preventive measures (including wearing masks and maintaining physical distance) may be necessary for long‐term pandemic management.^32^

## 5 CONCLUSION

To conclude, this study has found that omicron infection in allo‐HSCT setting has caused more pneumonia and more reactivation in advanced infection pattern (including moderate, severe, and critical). In addition, the risk factors of the severity of omicron infection in patients after allo‐HSCT include age, cGVHD, and inflammatory reaction factors.

## AUTHOR CONTRIBUTIONS

**Zhihui Li:** Conceptualization; methodology; writing – original draft. **Lei Wang:** Methodology. **Qinlong Zheng:** Methodology. **Teng Xu:** Software. **Keyan Yang:** Investigation. **Xianxuan Wang:** Investigation. **Xiaopei Wen:** Investigation; software. **Caiyan Zhang:** Methodology; investigation. **Jingjing Wang:** Conceptualization. **Yanzhi Song:** Conceptualization; methodology. **Yongqiang Zhao:** Methodology; investigation. **Xiaoyu Zheng:** Methodology. **Tong Wu:** Conceptualization; writing – review and editing.

## CONFLICT OF INTEREST STATEMENT

The authors have stated explicitly that there are no conflicts of interest in connection with this article.

## ETHICS STATEMENT

All the procedures carried out in studies, which involved human participants followed the ethical standards of the Beijing GoBroad Boren Hospital ethical committee and with the 1964 Helsinki declaration and its later amendments or comparable ethical standards (ethical approval number: KY2023‐001‐002).

## CONSENT TO PARTICIPATE

The consent was acquired from all participants.

## CONSENT FOR PUBLICATION

Consent for publication was acquired from all authors.

## Data Availability

All the data are involved in this study. Any data issues can be dealt with through contacting the email of the corresponding author.
